# Primary Human Osteoblasts Cultured in a 3D Microenvironment Create a Unique Representative Model of Their Differentiation Into Osteocytes

**DOI:** 10.3389/fbioe.2020.00336

**Published:** 2020-04-24

**Authors:** Gabriele Nasello, Pilar Alamán-Díez, Jessica Schiavi, María Ángeles Pérez, Laoise McNamara, José Manuel García-Aznar

**Affiliations:** ^1^Multiscale in Mechanical and Biological Engineering (M2BE), University of Zaragoza, Zaragoza, Spain; ^2^Biomechanics Section, Department of Mechanical Engineering, KU Leuven, Leuven, Belgium; ^3^Mechanobiology and Medical Device Research Group (MMDRG), National University of Ireland Galway, Galway, Ireland

**Keywords:** bone-on-a-chip, osteoblast differentiation, microfluidics, osteocyte, primary human cells, dendrite formation, *in vitro* bone model

## Abstract

Microengineered systems provide an *in vitro* strategy to explore the variability of individual patient response to tissue engineering products, since they prefer the use of primary cell sources representing the phenotype variability. Traditional *in vitro* systems already showed that primary human osteoblasts embedded in a 3D fibrous collagen matrix differentiate into osteocytes under specific conditions. Here, we hypothesized that translating this environment to the organ-on-a-chip scale creates a minimal functional unit to recapitulate osteoblast maturation toward osteocytes and matrix mineralization. Primary human osteoblasts were seeded in a type I collagen hydrogel, to establish the role of lower (2.5 × 10^5^ cells/ml) and higher (1 × 10^6^ cells/ml) cell density on their differentiation into osteocytes. A custom semi-automatic image analysis software was used to extract quantitative data on cellular morphology from brightfield images. The results are showing that cells cultured at a high density increase dendrite length over time, stop proliferating, exhibit dendritic morphology, upregulate alkaline phosphatase (ALP) activity, and express the osteocyte marker dental matrix protein 1 (DMP1). On the contrary, cells cultured at lower density proliferate over time, do not upregulate ALP and express the osteoblast marker bone sialoprotein 2 (BSP2) at all timepoints. Our work reveals that microengineered systems create unique conditions to capture the major aspects of osteoblast differentiation into osteocytes with a limited number of cells. We propose that the microengineered approach is a functional strategy to create a patient-specific bone tissue model and investigate the individual osteogenic potential of the patient bone cells.

## 1. Introduction

The comprehension of biological mechanisms in bones has a pivotal role in the development of successful clinical treatments. The developing field of bone engineering aims to take advantage of the innate repair capacity of this tissue (O'Brien, 2011), but the variability in the outcome of the products is one of the main limitations for their clinical translation. For example, the individual heterogeneous response in newly formed bone tissue formation leads to drastic changes in the scaffold design (Reznikov et al., 2019). *In vitro* models can explore the impact of individual response in tissue engineering products, but they require a bone cell source representing the phenotype variability.

Osteoblasts experience marked transitional stages during bone formation, involving changes in cell morphology and gene expression. Osteoblasts express ALP to provide phosphate ions and initiate the mineralization process (Chai et al., 2012). They also secrete osteocalcin (OCN), bone sialoprotein 2 (BSP2), and osteopontin (OPN) until the end of the mineralization phase (Franz-Odendaal et al., 2006). When osteoblasts turn to a more mature phenotype, they reduce ALP expression, become embedded in a mineralized matrix and form an interconnected network of osteocytes (Boukhechba et al., 2009). During this transition, osteoblasts upregulate characteristic proteins as E11 and dentin matrix protein 1 (DMP1) (Atkins et al., 2011). The expression of sclerostin (Sost gene) is associated with the final stage of osteocyte differentiation (Bonewald, 2011; Prideaux et al., 2016). However, osteoblasts can have three other possible fates but the mechanism regulating this transition is not clearly understood yet: they can become bone-lining cells (inactive osteoblasts), undergo apoptosis, or transdifferentiate into chondroid-depositing cells (Dallas and Bonewald, 2010). Gene expression profiles (Boukhechba et al., 2009; Sun et al., 2017) and immunohistochemistry stainings (Uchihashi et al., 2013; Sun et al., 2015; McGarrigle et al., 2016) in traditional 3D culture systems showed that the expression of osteoblast and osteocyte markers *in vitro* corresponded to the *in vivo* expression at the same differentiation stages (Franz-Odendaal et al., 2006).

In this context, *in vitro* bone tissue models are a prerequisite tool for answering specific questions of cell biology, where minimal platforms are mandatory for effective research on human tissue function (Wittkowske et al., 2016; Pirosa et al., 2018; de Wildt et al., 2019). While traditional tissue engineering aims to recapitulate whole organs *in vitro*, organ-on-chip systems “combines the key features of specific tissue microenvironments and architecture within a microfabricated device, facilitating the creation of 3D models that exhibit functional hallmarks of native tissues” (Zhang et al., 2018). They provide minimal units mimicking specific features of living organs and human physiology, as the tissue barrier properties of the human gut and lung, the parenchymal function of cardiac and hepatic tissue, the multiorgan interactions between the lymph node and the skin (Huh et al., 2011; Osaki et al., 2018; Ronaldson-Bouchard and Vunjak-Novakovic, 2018; Zhang et al., 2018).

Bone models on a chip were developed in recent years to reveal different elements of bone biology, each one based on a critical advantage of microengineered devices over traditional 2D or 3D macroscale *in vitro* systems. For example, the use of optically transparent materials allowed the monitoring of osteoblast motility in a confined 3D environment (Movilla et al., 2018). The results of this study elucidated the effect of ECM degradation and its architecture on osteoblast migration, by applying growth factor gradients or interstitial fluid flow (Del Amo et al., 2018). Moreover, the culture chamber geometries facilitate the reproduction of 3D organ-level structures. Microengineered devices highlighted how a 3D microvasculature integrates with the mineralized bone tissue microenvironment and enhances osteogenic differentiation of cells in the surrounding tissue construct (Bertassoni et al., 2014; Jusoh et al., 2015). Organ function relies on the presence of biomechanical and biochemical stimuli. Mechanical, electrical, and chemical stimuli can simultaneously stimulate cells cultured in organ-on-chip systems (Zhang et al., 2018). The use of compartmentalized culture environments promotes the selective application of those stimuli to different cell types. A 2D microfluidic platform with osteoclasts and osteocytes cultured in separate compartments was key to observe the cross-talk between mechanically stimulated osteocytes, osteoclast precursors and unstimulated osteocytes (You et al., 2008; Middleton et al., 2017). In general, microfabrication techniques applied to cell biology aims to develop advanced human disease models by the inclusion of pathological factors. For example, organ-on-a-chip systems with an *ex vivo* decellularized bone matrix or an osteo-cell conditioned extracellular matrix (ECM) have recreated the interplay between cancer and endothelial cells with the bone matrix in metastatic colonization (Bersini et al., 2014; Marturano-Kruik et al., 2018). Such systems consisted of a bone-like microenvironment including a monolayer of endothelial cells, which provided the capacity to obtain quantitative data on the extravasation of breast cancer cells. They were proposed as an advanced model to screen novel organ-specific therapeutics, since the osteo-cell conditioned microenvironment secreted specific chemokines affecting the extravasation process (Bersini et al., 2014).

Osteocytes are the central regulators of bone homeostasis *in vivo*. Their regulatory activity on both processes of bone formation and resorption relies on the secretion of specific proteins to interact with osteoblasts and osteoclasts (Bonewald, 2011). Mature osteocytes are the only bone cells expressing sclerostin after matrix mineralization, which suppresses osteoblast activity and inhibits further bone formation (Poole et al., 2005). On the other hand, the receptor activator of nuclear factor-kB ligand (RANKL) is a cytokine that controls osteoclastogenesis and has a fundamental role in bone remodeling. The expression of RANKL by osteocytes is ten times higher than by osteoblasts in normal mice and its specific deletion in osteocytes induces osteopetrosis (Nakashima et al., 2011).

The effect of osteocyte activities to other bone cells is a vital function that requires advanced *in vitro* models to be investigated. Traditional *in vitro* cultures replicated essential features of the interaction between osteoblasts and osteocytes, such as osteoblast maturation and matrix mineralization. These systems consisted of 3D collagen hydrogels/sponges and human osteoblast-like cells (Atkins et al., 2009; Bernhardt et al., 2019; Skottke et al., 2019 ) or mouse clonal cell-lines (MC3T3-E1 and IDG-SW3) (Woo et al., 2011; McGarrigle et al., 2016), with the last ones usually preferred due to the excessive number of cells required. On a micro-scale, a 2D layer of MC3T3-E1 cells spontaneously formed a mineralized collagenous matrix in long-term cultures (up to 1 month) (Hao et al., 2018). Cells were mainly present in the apical and basal layers of the matrix, possibly due to excessive proliferation of the mouse cell-line. No previous bone-on-a-chip device has differentiated primary human osteoblasts into osteocytes while embedded in a 3D extracellular matrix. There is a need for a novel microengineered model to explore the interaction between the two cell types.

Microfabricated platforms facilitate the use of cells derived from patients, thus creating models of human physiology with higher clinical impact. Primary and mature human cells are difficult to extract in large amounts, proliferate slowly and tend to dedifferentiate rapidly. Those systems require a limited amount of cells and aim to recreate the native microenvironment: they provide an approach to culture cells representing the variability of the cell phenotype (Zhang et al., 2018). This is a key advantage of the organ-on-a-chip over traditional technologies, considering the growing concern about the use of immortalized cell-lines for reliable human models (Lorsch et al., 2014). Cell-lines could lead to different results with respect to primary cells from the same species. For example, murine early osteocyte cell-line MLO-A5 created an interconnected 3D network in a microfluidic perfusion device (Gu et al., 2015) but produced a highly mineralized and dense tissue compared to primary murine bone cells (Sun et al., 2015).

We developed a bone-on-a-chip device to translate the findings of traditional macro-models on differentiated osteocyte networks to the scale of micro-devices. We hypothesized that the combination of primary human cells and a 3D fibrous collagen matrix in a microengineered platform creates a more robust model to recapitulate osteoblast maturation toward osteocytes and matrix mineralization. We developed a 3D cell culture system where the structural and biochemical microenvironment induced (1) the mineralization of a collagen matrix and (2) the differentiation of primary human osteoblasts into osteocytes. The specific aims were to determine the osteogenic behavior of osteoblasts embedded in a collagen type I hydrogel in terms of: (1) cellular morphology, (2) dendrite tracking, (3) cell proliferation, (4) ALP activity, (5) calcium deposition, and (6) synthesis of specific osteoblasts and osteocytes markers, respectively, BSP2 and DMP1, as well as the role of cell density in the formation of a bone tissue model.

## 2. Materials and methods

### 2.1. Cell culture

The low quantity needed for bone-on-a-chip experiments and the stability in the expression of the most critical genes during *in vitro* culture (Sun et al., 2017) favored the choice of primary human osteoblasts (HOBs) for the bone tissue model presented in this study.

HOBs were purchased from PromoCell (C-12720, Germany). These cells are fully differentiated osteoblasts isolated from femoral trabecular bone tissue from the knee or the hip joint region of healthy single donor. HOBs from two different donors were used (donor 1: caucasian female 86 years, donor 2: caucasian male 74 years). Cells were cultured under standard conditions (5% CO_2_, 37°C), in cell expansion medium containing standard Dulbecco's Modified Eagle Medium (DMEM low glucose; Thermo Fisher Scientific, MA) supplemented with 10% fetal bovine serum (FBS; Thermo Fisher Scientific), 100 U/mL penicillin, 100 μg/mL streptomycin, and 2 mM L-glutamine (all Lonza, Switzerland). At each passage, cells were washed with phosphate buffered saline (PBS; Lonza), detached with TrypLE^*TM*^ Express (Invitrogen, CA) and plated in T25 cell culture flasks (Thermo Fisher Scientific) at a density of 15,000 cells/cm^2^. Cells were used at passage 4–7 and mixed to the collagen gel solution. Final cell densities of 2.5 × 10^5^ (low) and 1 × 10^6^ cells/ml (high) were based on recent findings on osteocyte differentiation in traditional *in vitro* systems (McGarrigle et al., 2016).

Once loaded to the organ-on-a-chip devices, HOBs were cultured under static conditions in cell osteogenic medium, containing cell expansion medium supplemented with 50 μM ascorbic acid and 10 mM β-glycerol phosphate (both Sigma-Aldrich). Those supplements are widely accepted to induce osteogenic differentiation of stem cells *in vitro* and deposition of mineralized extracellular matrix (Langenbach and Handschel, 2013).

### 2.2. Bone-on-a-Chip System

**Figure 1:**
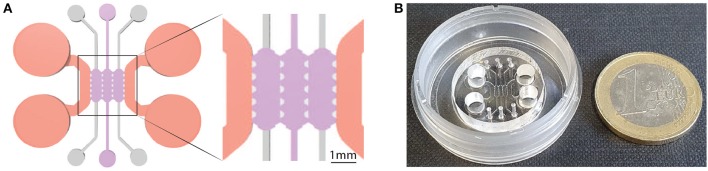
Geometry of the bone-on-a-chip system. **(A)** 2D top view of the bone-on-a-chip system and detail of the culture chamber divided into three compartments by trapezoidal columns. The cell laden collagen hydrogels (purple) were loaded through the central compartment, while cell culture media (pink) was provided through the lateral channels. The height of the culture chamber is 290 ± 20 μm. **(B)** Picture of the bone-on-a-chip system bonded to a 35 mm glass.

Bone-on-a-chip devices were fabricated in poly(dimethlysiloxane) (PDMS) by soft lithography, following the methodology described by Shin et al. (2012). A commercial product was used to produce the silicone elastomer (Sylgard 184 Silicone Elastomer Kit, Dow Chemical, Germany), which comprises a polymeric base and a silicone resin solution as curing agent. The two liquid parts were mixed in a 10 (base) :1 (curing agent) ratio and poured in a master made of SU-8 where the microengineered geometry was patterned with a photolithography technique. The masters were then placed in a vacuum desiccator for one hour, to remove air bubbles in the PDMS solution, and kept in a dry oven overnight to cure the mixture. Later, dermal biopsy punches were used to create the reservoirs for cell culture media and the hydrogel inlets to the culture chamber (Figure 1A). PDMS devices followed a wet and a dry autoclave cycle before being bonded to a 35 mm glass (Ibidi, Germany) by plasma treatment (PDC-32G Basic Plasma Cleaner, Harrick Plasma, NY, USA) under vacuum conditions (Figure 1B). They were then coated with PDL (poly-D-lysine; 1 mg/ml in phosphate buffered saline; Sigma-Aldrich, Germany) and washed after 4 h to enhance matrix adhesion. Before use, the devices were left in a dry oven at 80°C for 48 h to restore the hydrophobicity of the bonded surfaces (Shin et al., 2012).

A previously developed geometry that consists of a central culture chamber divided into three compartments was selected for this study (Del Amo et al., 2017). Briefly, the length of a single compartment was 2.5 mm, the width was 1.145 mm, and the average height was 290 μm (Figure 1A). The chip uses equally spaced (175 μm) trapezoidal columns to separate the compartments and support the hydrogel via surface tension: gel contraction occurs only on the lateral channels while the 3D culture environment is maintained in the central channel during the whole experiment.

Cell laden collagen hydrogels, with a final collagen concentration of 6 mg/ml, were prepared by mixing in an ice bath collagen type I solution (Rat Tail; 8.9 mg/ml; Corning, NY), 10x Dulbecco's phosphate buffered saline (DPBS; Sigma-Aldrich), 0.5 M NaOH (Sigma-Aldrich) to adjust the pH to 7.4–7.6, and human osteoblasts suspended in cell expansion medium. After gently pipetting the solution into the culture chamber, it polymerized for 20 min in a humid chamber at 37°C.

The temperature and the pH conditions mentioned above induced a self-assembled gelation process of the collagen hydrogels, where collagen fibers are physically crosslinked (Chuang et al., 2018). During the polymerization process, collagen fibers create an interpenetrating polymer network in presence of living cells (Rowe and Stegemann, 2006). The microarchitecture and the mechanical properties of the collagen fibrous hydrogels used in this study were characterized in previous studies (Moreno-Arotzena et al., 2015; Del Amo et al., 2018). In the present work, we selected the 6 mg/ml gel because its Young modulus (~0.72 kPa, from Valero et al., 2018) was comparable to the one (~0.58 kPa) that allowed a homogeneous cell distribution in a 3D matrix and enhanced osteocytic differentiation of MC3T3-E1 cells (McGarrigle et al., 2016).

### 2.3. Phalloidin and DAPI Fluorescence Staining

Morphological analysis of cell phenotype is commonly used to assess osteoblast-osteocyte differentiation (Mullen et al., 2013). Cells were fixed after 21 days of culture using 4% (w/v) paraformaldehyde (PFA, Thermo Fisher Scientific) for 30 min, blocked overnight in PBS with 5% bovine serum albumin (BSA) and incubated overnight with Phalloidin-TRITC (0.1 mg/ml in PBS, Sigma Aldrich) and DAPI (0.01 mg/ml in PBS, Invitrogen). Samples were observed with a confocal microscope (Zeiss LSM880, Zeiss, Germany). Maximum intensity and 3D volumetric images were generated from z-stacks using ImageJ software (v 1.49, NIH).

### 2.4. Cell Dendrite Tracking

Brightfield images of the same samples were taken to track changes in dendrite morphology. Devices were observed at 10x and 20x magnification over the 21 days of culture with an inverted brightfield microscope (Nikon D-Eclipse C1, Japan) and focused in the middle of the culture chamber. Z-stacks were obtained with a distance of 5 μm between each slice and for a maximum thickness of 100 μm. A minimum number of 3 stacks were taken for each cell density at each timepoint.

A custom semi-automatic image analysis software was developed with Python programming language to track cell dendrite length over time. Original z-stacks images were opened with the Bio-format software tool (Linkert et al., 2010) and processed with the scikit-image library (van der Walt et al., 2014). After cells were manually contoured, an automatic algorithm computed the medial axis transform of the binary cell mask. An Otsu's thresholding algorithm later separated the cell body primary and secondary dendrites from the cell body. The software quantified the length of cell primary protrusions, defined as the longest branches starting from the cell body, and detected cell-cell connections if cell boundaries were in the same image of the z-stack.

Osteocytes exhibit exploratory dendrites that extend and retract from the surrounding extracellular matrix before forming a fully developed osteocyte network (McGarrigle et al., 2016). Cell primary protrusions longer than 10 μm were defined as dendrites, as previously described (Mullen et al., 2013).

### 2.5. DNA Content

Osteoblasts move from a proliferative (preosteoblast) to a quiescent phase (mature osteoblasts/osteocytes) during differentiation (Franz-Odendaal et al., 2006). Thus, DNA content was measured using the Hoechst 33,258 DNA assay to monitor cell proliferation over time. Cells were isolated by digesting the collagen matrix overnight in a solution of collagenase from *Clostridium histolyticum* (Sigma-Aldrich, 2 mg/ml, ≥ 125 CDU/mg) and centrifugating cell suspension at 13,000 rpm for 15 min. 100 μl of Hoechst buffer [1 mM EDTA, 10 mM Tris (hydroxymethyl) aminomethane and 0.1 M Sodium Chloride at pH 7.4, all reagents from Sigma-Aldrich] was added to the pellet. Cells were lysed by applying 3 cycles of freezing (−80°C)-thawing procedure before running the biochemical assay. Later, 20 μl of cells lysate or DNA standards were suspended in 200 μl of Hoechst dye solution (0.1% v/v, Sigma-Aldrich) and added in a 96-well plate in triplicate. Fluorescence was then measured (excitation: 380 nm; emission: 440 nm) using a fluorescence spectrophotometer (Synergy HT Multi-mode microplate reader, BioTek Instruments, VT, USA). Readings were converted to DNA content using a standard curve, according to the manufacturer's protocol, with samples containing no cells subtracted as background.

### 2.6. Extracellular ALP Activity

The metalloenzyme ALP initiates the calcification process by providing inorganic phosphates (Coleman, 1992; Golub and Boesze-Battaglia, 2007). It is used as a marker for osteoblast activity, since ALP expression changes over time when osteoblast differentiation occurs (Atkins et al., 2009). Extracellular ALP activity was measured using a colorimetric assay of enzyme activity (SIGMAFAST p-NPP Kit, Sigma Aldrich). It uses p-nitrophenyl phosphate (pNPP) as colorimetric substrate that changes absorbance when dephosphorylated by ALP. Cell culture media was changed and sampled after 2 h at days 3, 7, 14, 21, and stored at −80°C. After thawing, 40 μl of medium were added to a 96-well plate in triplicate with 50 μl of pNPP solution (Birmingham et al., 2012). Samples were incubated at room temperature in the dark for 1 h, and absorbance was read at 405 nm with a spectrophotometer. Readings were converted to ALP production using a standard curve, with samples containing no ALP subtracted as background. ALP production was normalized by the DNA content of each sample in order to get comparable estimations of ALP activity between samples with different initial cell seeding density.

### 2.7. Mineralization

Calcein green staining (Sigma Aldrich) was performed to analyze calcium deposition without affecting cell viability after 7, 14, and 21 days (Schiavi et al., 2015). Calcein was dissolved in 0.5 M NaOH solution (Sigma Aldrich) at 2.5 mg/ml. After mixing, the solution was sterilized with a 0.2 μm Nylon filter, wrapped with aluminum foil and stored at 4°C. Cells were incubated with calcein (25 μg/ml in osteogenic media) (Sigma) for 5 days, washed 3 times in PBS and fixed using 4% (w/v) PFA. Samples were imaged with a confocal microscope (λ_*ex*_ = 470–509 nm) and maximum intensity images were generated from z-stacks using ImageJ software.

### 2.8. Immunofluorescent Staining

BSP2 is a non-collagenous protein upregulated by osteoblasts during the tissue mineralization and downregulated when osteocyte differentiation occurs. DMP1 is an extracellular matrix protein associated with osteocytes. BSP2 and DMP1 immunofluorescent stainings indicate whether cells cultured in the bone-on-a-chip samples synthetized osteoblast or osteocyte markers, respectively (Atkins et al., 2009; Dallas and Bonewald, 2010).

Cells were fixed after 7, 14, 21 days of culture using 4% (w/v) PFA for 30 min and blocked overnight with 5% BSA. Later, samples were incubated at 4°C overnight with mouse monoclonal BSP2 (Santa Cruz Biotechnology, sc-73630, Texas) or DMP1 (Santa Cruz, sc-73633) antibody at a dilution of 1:100 in PBS with 0.5% BSA. After washing 3 times for 5 min with PBS, samples were incubated at 4°C for 6 h with the relevant secondary antibody (for BSP2, goat anti-mouse Alexa Fluor® 555, Molecular Probes, Oregon, A21424; for DMP1, goat anti-mouse Alexa Fluor® 633, Molecular Probes, A21052) at a dilution of 1:50 in PBS with 0.5% BSA. Cell nuclei were then counterstained with DAPI and samples were observed with a confocal microscope. Maximum intensity images were generated from z-stacks using ImageJ software.

### 2.9. Statistics

All experiments were conducted in technical triplicates (*n* = 3) with two independent experiments, by using different donors. Python programming language was used to run all the statistical analyses. We used one-way analysis of variance (ANOVA) for all the biochemical analyses to assess significant differences between timepoints, followed by pair-wise multiple comparison procedure (Tukey's HSD test). Linear regression models described the variation of dendrite length over time. Experimental data are presented as mean ± 95% of confidence interval if not otherwise specified. *P*-value < 0.05 was considered significant.

## 3. Results and Discussion

Recent advances in mimicking bone with microengineered platforms are reducing the gap between the tissue and other more developed organ-on-chip models but, to our knowledge, they did not reach to show the development of a device with differentiated osteocytes and a 3D mineralized matrix.

Given that primary human osteoblasts embedded in a 3D fibrous collagen matrix is an optimal environment to encourage osteocyte differentiation (Atkins et al., 2009; Woo et al., 2011; McGarrigle et al., 2016), we hypothesized that the same environment translated to organ-on-a-chip scale recapitulates two key *in vivo* bone properties: osteoblast maturation and matrix mineralization. To determine the development of mature bone formation, we investigated the major aspects underlying osteoblast-osteocyte differentiation: cell morphology variation, mineralization, and protein synthesis.

### 3.1. Cells Show Dendritic Morphology While Increasing Primary Dendrite Length Over Time

The osteoblast-osteocyte differentiation is a complex mechanism, still under investigation, with relevant changes in cell morphology, polarization, cytoplasmic volume, number of cell organelles, proliferation, gene expression, type of collagen deposited, and bone deposition rate. During these transitional stages, the extension of cellular dendrites or pseudopodia is the one of most recognizable variations (Franz-Odendaal et al., 2006).

**Figure 2:**
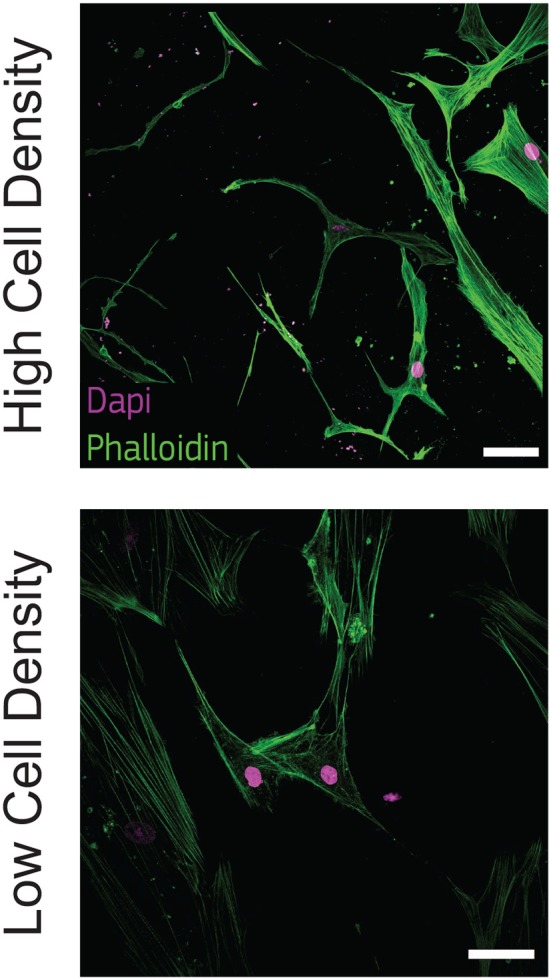
Cells cultured at high density show dendritic morphology. Representative confocal images of cell nuclei (DAPI) and actin filaments (phalloidin) after 21 days of culture in bone-on-a-chip samples at high and low cell density. Scale bar, 50 μm.

To observe this morphological change, we stained actin filaments at the latest timepoint (21 days) and we qualitatively observed that cells at high density exhibited a dendritic morphology as well as initial interconnections. On the other hand, cells at low density kept an aligned morphology with a limited amount of dendrites (Figure 2).

Osteoblasts were already classified with a spread, aligned, transitional, or dendritic morphology during their differentiation into osteocytes *in vitro* (Mullen et al., 2013), and mineralizing conditions enhanced the development of a stellate morphology (Atkins et al., 2009). Microfluidics showed that pre-osteoblastic mouse cells adhere and spread differently along a stiffness gradient, but it did not highlight any morphological variation (Almodóvar et al., 2013). Here, the difference in cell shape suggests that human osteoblasts cultured at 1 × 10^6^ cells/ml (high density) are more differentiated into osteocytes than cells cultured at 2.5 × 10^5^ cells/ml (low density) (Figure 2). The dendritic morphology for the high density group reported here is consistent with the results in a traditional 3D *in vitro* systems, where mouse bone cells cultured at a low cell density (2.5 × 10^5^ cells/ml) had a lower percentage of dendritic cells with respect to cells cultured at high density (2 × 10^6^ cells/ml) (McGarrigle et al., 2016). Dendritic protrusions of primary human osteoblasts in collagen hydrogels is documented in the literature for initial cell seeding densities lower than 2.5 × 10^5^ cells/ml (Atkins et al., 2009; Bernhardt et al., 2019; Skottke et al., 2019 In these cases, the maximum collagen concentration in the hydrogel was 3 mg/ml, which is half of the 6 mg/ml used in our study. Hydrogels with lower collagen concentration have lower mechanical properties (Chuang et al., 2018), which favor a dendritic cell morphology even at lower cell seeding densities (McGarrigle et al., 2016). Osteoblast differentiation was reduced in hydrogels with low collagen concentration when they were modified with biomimetically or strontium-doped mineralized collagen, confirming that the higher stiffness of the matrix delays the differentiation for low cell seeding densities (Bernhardt et al., 2019). On the other hand, hydrogel contraction limits the use of high cell seeding densities. Our bone-on-a-chip system, where trapezoidal columns within the culture chamber reduces the hydrogel contraction, facilitated the use of hydrogels with higher collagen concentration, thus favoring the culture of cells at higher seeding densities and the exhibition of a dendritic morphology at higher collagen concentrations.

**Figure 3:**
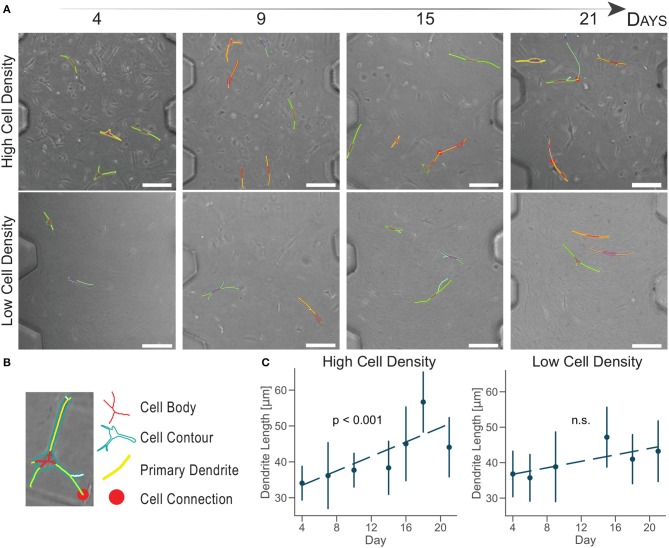
Cells cultured at high density increase primary dendrite length over time. **(A)** Brightfield images of cultured human osteoblasts with superimposed tracking. Scale bar = 150 μm. **(B)** Cell image detail. Identification of the cell body, contour, primary dendrites, and connection with a semi-automatic algorithm. **(C)** Primary dendrite length measured with a customed software for dendrite tracking. Linear regression model in dashed lines (*n* ≈ 50 cells per timepoint). Data are mean ±95% c.i.

Experimental biology requires novel automatic tools for image analysis to support the conversion of microscopy images into quantitative data (Eliceiri et al., 2012). To demonstrate a consistent change in cellular morphology, a customed image analysis software was developed to automatically separate cell body from dendrites, after a manual selection of the cell boundary (Figure 3B). The software quantified the cell dendrite length and detected cell-cell connections of cells in the same image of the z-stack (Figure S1, Video S1).

Cells cultured at high density experiences a significant dendrite growth of 1.01 ± 0.25 μm/day (Figure 3C) over the whole period of culture, confirming osteoblast change to a dendritic morphology compared to the lower density.

Cells cultured at low density did not increase dendrite length over time (Figure 3C). This analysis did not show a quantitative change in cell morphology for the low density group, as suggested by the aligned shape observed in confocal imaging (Figure 2).

Compared to traditional *in vitro* culture, the ratio between the volumes of culture and the fewer number of cells used in microengineered devices make quantitative image analyses more representative of the whole cell population present in each sample. The number of cells tracked in this study (*n* ≈ 50 cells per timepoint) is 100 times lower than the total amount of cells in our samples (≈5,000 cells), while in traditional systems it is usually 1,000,000 times lower. Moreover, the high control of the geometries of the organ-on-a-chip culture chamber allows the identification of the same image location at each timepoint. The combination of the organ-on-chip technology with a semi-automatic software that decreases operator-dependent errors produce a more accurate and reliable platform for quantitative image analysis.

The evaluation of cell-cell connections showed a limited variation over time, due to the requirement for cell boundaries to be on the same image of the z-stack (Video S1). However, we observed a slight increase in cell connections for cells cultured at high density over time (Figure 3A). Osteoblasts did not form an interconnected network, they rather showed exploratory dendrites to create transient connections and position themselves in the extracellular matrix (Zhang et al., 2006; McGarrigle et al., 2016). The dendritic morphology at the end of the culture and the increase in exploratory dendrite length indicated that osteoblasts cultured at high density underwent differentiation into an early osteocytic phenotype.

### 3.2. Alkaline Phosphatase (ALP) Upregulation Is Associated With the Interruption of Cell Proliferation

The cessation of cell proliferation and collagen matrix production follows the secretion of soft osteoid that surrounds osteoblasts initiating differentiation (Atkins et al., 2011). To determine whether the culture microenvironment regulated cell proliferation in our bone-on-a-chip, we degraded the 3D collagen matrix, extracted cells and quantified the evolution of the DNA content.

**Figure 4:**
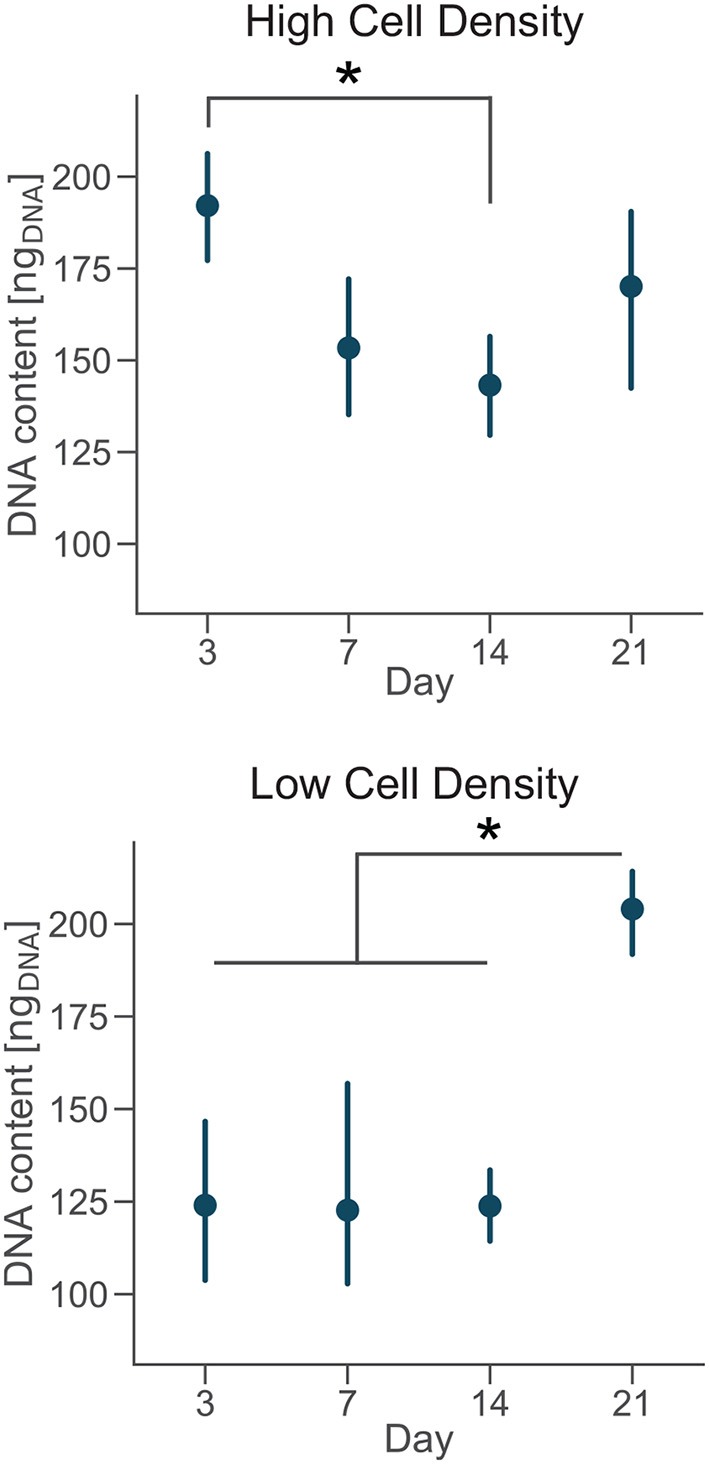
Cells cultured at low density proliferate over time. DNA content of each group at day 3, 7, 14, and 21. Point plots denote the mean of *n* = 6 samples with error bars representing 95% of confidence interval. *Represents statistical significance (*p* < 0.05) between indicated groups using one-way ANOVA with Tukey's *post-hoc* test.

DNA content had a negative trend over time for the high cell density group, as suggested by the lower DNA content at day 14 compared to day 3 (Figure 4). The amount of DNA measured from a unique cell type is proportional to the total number of cells. Thus, osteoblasts were not proliferating in our bone-on-a-chip system, plausibly because they were undergoing a differentiation process. Only a limited proportion of osteoblasts differentiates into osteocyte *in vivo* while the rest of the population follows other fates, including apoptosis (Franz-Odendaal et al., 2006). If cells seeded at high density were differentiating into osteocytes, the decreasing trend in the DNA amount confirmed that our microenvironment also induced the apoptosis of a subpart of the osteoblast population.

A 3D culture environment made of a microbeads assembly already showed that osteoblastic cells cultured in a microfluidic system stop proliferating, compared to a control group cultured in a plate where cells were proliferating. The cell number quantification in the microbead assembly was estimated by the ratio of the number of cells and the number of microbeads imaged (Sun et al., 2017). Here, we developed a protocol to get a more direct measure of the amount of cells over time in the microengineered sample.

In the low cell density group, by day 21 the DNA content was significantly higher than early timepoints (Figure 4), suggesting that cells started proliferating under these culture conditions. Our results show that after an initial period of quiescence, osteoblasts seeded at low density maintained their proliferative capacity in our bone-on-a-chip, supporting the hypothesis that they were not differentiating into osteocytes. The use of primary human cells was crucial to capture the difference between proliferative and non-proliferative conditions in the bone-on-a-chip model. Cell-lines have high proliferation rates which make them not very suitable to study this precise aspect of bone cells function (Clover and Gowen, 1994). For example, a mouse osteoblast cell-line (MC3T3-E1) showed to proliferate even if cells were differentiating into osteocytes and creating an interconnected network between dendritic cells (McGarrigle et al., 2016). The combination of primary human cells and microengineered technology give a valid solution to culture cells representative of the *in vivo* phenotype maximizing the number of samples. Such system is a unique representative model of osteoblast proliferation in a physiologic 3D environment.

The transition from a proliferative to a quiescent phase during osteoblast differentiation is concomitant to the upregulation of ALP and its later reduction (Boukhechba et al., 2009). To assess osteoblast secretion of ALP during culture, we quantified the evolution of enzyme activity in the culture media and the effect of cell density.

**Figure 5:**
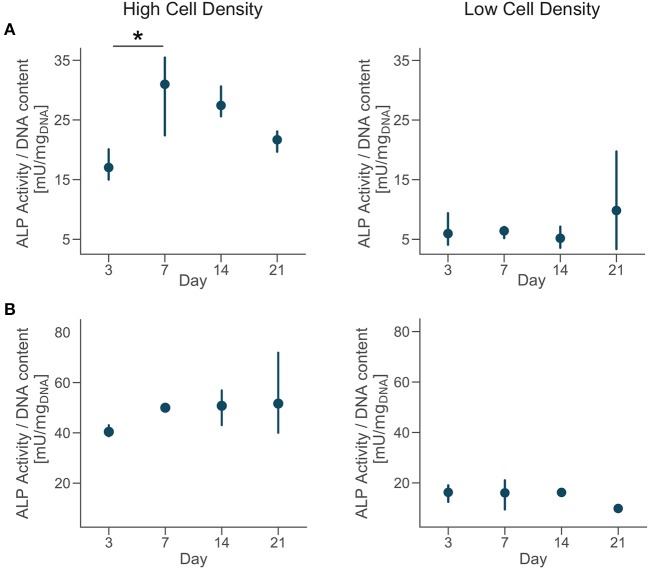
Cells cultured at high density upregulate alkaline phosphatase (ALP) activity. Extracellular ALP activity of each group at day 3, 7, 14, and 21. Point plots denote the mean of *n* = 3 samples (row A—donor 1, row B—donor 2) with error bars representing 95% of confidence interval. *Represents statistical significance (*p* < 0.05) between indicated groups using one-way ANOVA with Tukey's *post-hoc* test.

Extracellular ALP activity was normalized by the DNA content and showed an upregulation for the high density group compared to the low density in both donors (Figure 5). The ALP activity had increasing trend for cells cultured at high density, even if the magnitudes of the ALP measurements were higher for donor 2 compared to the donor 1. Moreover, cells from the donor 1 showed a significantly higher ALP activity by day 7 followed by a decreasing trend (Figure 5A). Differences in trends of the ALP measurements might be related to inter-donor variability, and more specifically to the differences in age and sex between donors. Cells from the younger male donor (donor 2) had higher absolute ALP activity and didn't show the trend observed in donor 1. However, the upregulation of ALP activity for cells cultured at higher density confirmed the higher potential for matrix mineralization and osteogenic differentiation for both donors. In tissue engineering, the upregulation of ALP expression relates to osteogenesis and is a precursor to mineralization of the tissue (Golub and Boesze-Battaglia, 2007). Previous studies in microfluidic devices only showed the upregulation of ALP activity at the end of the culture, proving that those platforms promoted osteogenesis (Leclerc et al., 2006; Jang et al., 2008; Hao et al., 2018). Here, we measured the temporal evolution of ALP activity during the whole culture and it suggested that osteoblasts cultured at high density had a phase of increase of mineralization in the first week of culture and later continued the differentiation process.

Calcein is a fluorochrome binding calcium at bone mineralization front (Yeh et al., 2018), thus identifying newly mineralized matrix. Calcium staining with calcein at days 7, 14, and 21 showed that primary human osteoblasts mineralized the extracellular matrix over the whole period of culture. Images qualitatively confirmed the results of ALP activity, with a higher presence of calcium ions for the cells cultured at high density (Figure S2). Our results suggest that the low cell density tested (2.5 × 10^5^ cells/ml) was insufficient to initiate osteoblasts differentiation in this hydrogel and they needed to proliferate more before undertaking this pathway.

### 3.3. Cell Density Regulates Osteoblast/Osteocyte Marker Synthesis

Osteoblasts transitional stages during bone formation involve changes in cell morphology and protein synthesis. As we found differences in cell morphology, protrusion length dynamics, proliferation, and osteogenesis, we investigated the qualitative expression of bone matrix proteins. We selected two specific proteins BSP2 and DMP1, representative of osteoblast and osteocyte phenotype respectively, to determine the effect of cell density on their production.

**Figure 6:**
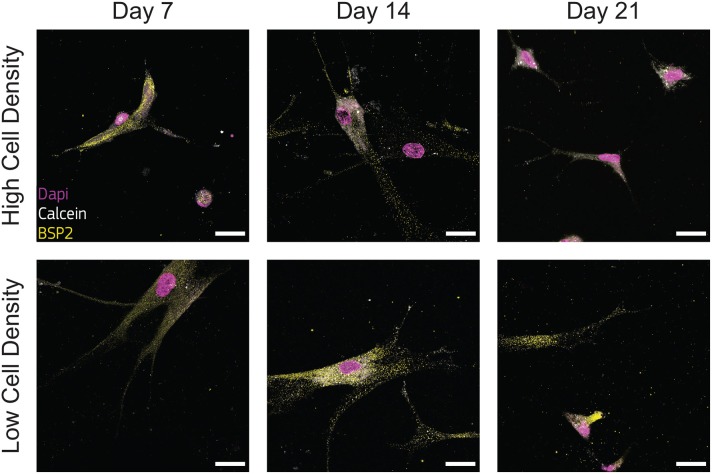
Cells cultured at low density synthesize bone sialoprotein 2 (BSP2) at all timepoints. Representative confocal images of cell nuclei (DAPI), calcium ions (calcein), and BSP2 in bone-on-a-chip samples cultured at high and low cell density. Scale bar, 30 μm.

Immunofluorescent staining for BSP2 demonstrated positive synthesis within the proximity of cells in the earliest days of culture for both cell density groups, confirming the initial osteoblast phenotype of the cells used at the beginning of the experiment. Osteoblasts cultured at low density produced BSP2 at all timepoints (Figure 6), suggesting that no transition to osteocyte occurred for this group. On the contrary, the high density group reduced BSP2 expression at the end of the culture (21 days, Figure 6). The lower presence of BSP2 denoted that cells cultured at high density group changed differentiation stage in the bone-on-a-chip devices. BSP2 expression is characteristic of osteoblast phenotype, even if it was observed in lower amounts in osteocytes (Franz-Odendaal et al., 2006). Another osteocytic marker needs to determine whether cells of the high density group underwent this specific fate.

**Figure 7:**
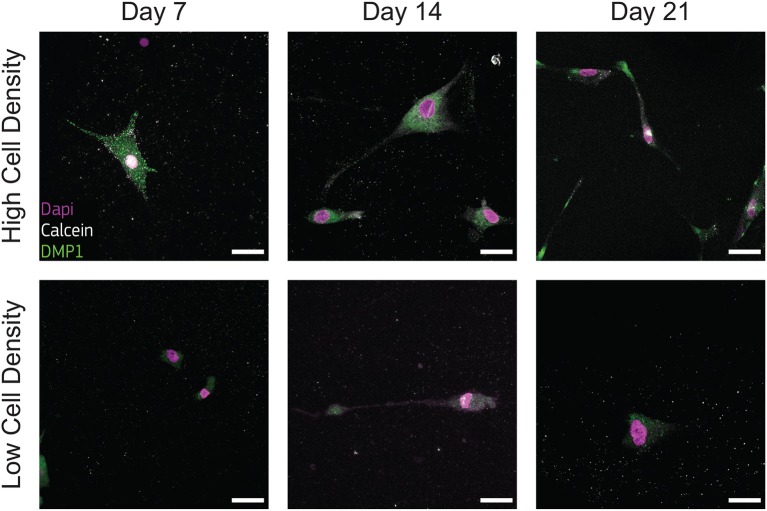
Cells cultured at high density synthesize dental matrix protein 1 (DMP1). Representative confocal images of cell nuclei (DAPI), calcium ions (calcein), and DMP1 in bone-on-a-chip samples cultured at high and low cell density. Scale bar, 30 μm.

Low presence of DMP1 staining were observed for cells cultured at low density (Figure 7), supporting that cells of this group experienced a limited transition into the osteocyte stage during the whole culture period. DMP1 was more expressed by the human osteoblasts cultured at high density at all timepoints (Figure 7). The faster osteoblasts transition to osteocytes was confirmed by the presence of this osteocytic marker, together with the decreased presence of the osteoblast marker BSP2 (Figure 6). The positive DMP1 staining for cells cultured at high density reported here is consistent with the results in a traditional 3D *in vitro* systems, where cells cultured at higher density (2 × 10^6^ cells/ml) produced more DMP1 as dispersed nodules within the surrounding matrix (McGarrigle et al., 2016).

### 3.4. Cells Cultured at Higher Density Differentiate Faster Into Osteocytes

**Figure 8:**
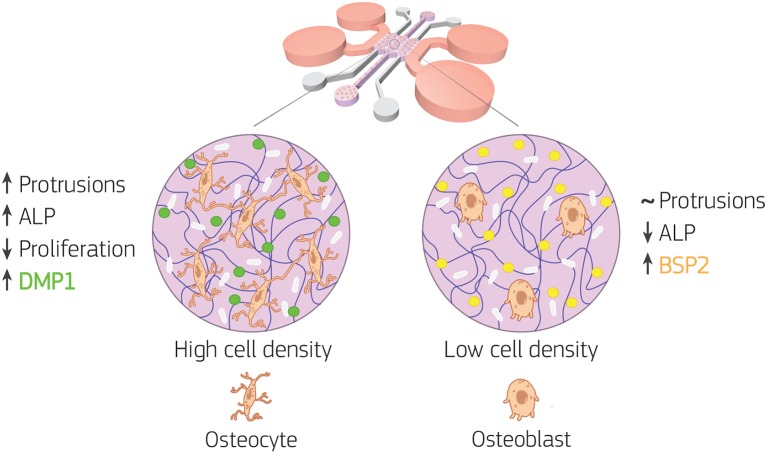
Bone-on-a-chip development with two cell seeding densities. Primary human osteoblasts were encapsulated in type I collagen hydrogel (purple in the figure) at 2.5 × 10^5^ (low cell density) and 1 × 10^6^ (high cell density) cells/ml, then loaded in the bone-on-a-chip devices and cultured up to 21 days to study matrix mineralization and cell differentiation into osteocytes. Cell morphology, dendrite tracking, DNA content, alkaline phosphatase (ALP) production, calcium deposition (white in the figure), and immunofluorescent staining of bone sialoprotein 2 (BSP2, yellow in the figure) and dentin matrix protein 1 (DMP1, green in the figure) were performed to assess the osteogenic phenotype of the cells. At low cell density, the biochemical 3D microenvironment induced only the expression of osteoblast markers (ALP, BSP2). While the change in cell morphology, the upregulation of ALP and the expression of DMP1 suggested that osteoblast transition to osteocytes occurred faster for cells cultured at high density.

Overall, our data showed how the 3D microenvironment in our bone-on-a-chip regulated osteoblast-osteocyte differentiation (Figure 8). The microengineered technology created a highly controllable environment to study the maturation of primary human bone cells in a 3D extracellular matrix made of the most abundant organic component in bone tissue. A custom semi-automatic image analysis software led to the extraction of quantitative data on the cellular morphology from microscopy images. In addition, the use of primary human cells at high cell density was crucial to mimic the physiological shift from proliferative to non-proliferative conditions during the differentiation process. The expression of characteristics osteoblast and osteocyte markers confirmed differentiation with changes in synthesis of extracellular matrix proteins. Furthermore, by 21 days of *in vitro* culture, osteoblasts cultured at high density increased the protrusion length over time, exhibited dendritic morphology, did not increase cell number, upregulated ALP activity, downregulated BSP2 synthesis and produced more DMP1. Thus they underwent changes in cell morphology, proliferation, mineral deposition and protein production that are specific of the differentiation into osteocytes. Conversely, osteoblasts cultured at low density had constant protrusion length up to 21 days, proliferated, had constant ALP activity and produced only BSP2. They consistently exhibited no transition toward the osteocytic phenotype.

One limitation of this study is the presence of a 3D hydrogel embedding cells in a narrow space that made RNA purification not possible after extraction for gene expression analysis. Moreover, the cell extraction protocol took one night for the complete digestion of the collagen matrix, which would inexorably affect results from PCR. Previous works on cell differentiation in microfluidic systems focused on the protein expression rather than gene profiles (Kim et al., 2011; Li et al., 2014; Park et al., 2015; Zhang et al., 2015; Agrawal et al., 2017), considering the technical challenges and that protein biosynthesis depends on transcript levels as well as other factors such as the local availability of resources (Liu et al., 2016). We believe that our results on dendritic morphology, cell proliferation, alkaline phsphatase activity and protein synthesis firmly indicate osteoblasts differentiation and the effectiveness of the microengineered technology used.

## 4. Conclusions

Traditional *in vitro* systems already showed that primary human osteoblasts embedded in a 3D fibrous collagen matrix differentiates into osteocytes under specific conditions. Here, we translated the findings of traditional macro-models on osteoblast differentiation to the organ-on-a-chip scale. We created a minimal functional unit that captures the major aspects underlying the differentiation of primary human osteoblasts into osteocytes, which depends on the cell seeding density. A custom image analysis software was developed to support the semi-automatic analysis of cell dendrite elongation. Only the human osteoblasts seeded at higher density underwent the change in cell morphology, proliferation, mineral deposition and protein synthesis that are specific of the differentiation into osteocytes. The use of the microengineered technology to study the maturation of primary human osteoblasts in a 3D fibrous extracellular matrix takes advantage of the limited number of cells required by these systems, creating a unique platform to represent the phenotype variability as well as to elucidate the role of single or simultaneous stimulation in osteoblast maturation. From a clinical perspective, the bone-on-a-chip presented in this work provides the minimal functional microenvironment to build patient-specific bone models to study the individual osteogenic potential and the effect of alternative therapies.

## Data Availability Statement

Raw data is available upon request to the corresponding author. The image analysis software used for cell protrusion tracking in this study was developed in Python 3, and is available under github.com/gabnasello/cellprotrusiontracker-frontiers2020.

## Author Contributions

GN, PA-D, and JS designed the experiments. GN and PA-D carried out the experiments. GN developed the image analysis software. GN, JS, MP, LM, and JG-A participated in study design. GN analyzed the results and wrote the manuscript. All authors discussed the results and approved the final version of the manuscript.

## Conflict of Interest

The authors declare that the research was conducted in the absence of any commercial or financial relationships that could be construed as a potential conflict of interest.
