# Supplementary material for: Primary Human Osteoblasts Cultured in a 3D Microenvironment Create a Unique Representative Model of Their Differentiation Into Osteocytes
Source: Front Bioeng Biotechnol. 2020 Apr 24;8:336. doi: 10.3389/fbioe.2020.00336 (PMC7193048; doi:10.3389/fbioe.2020.00336)
Supplement: Supplementary file 1 [file Data_Sheet_1.PDF]

# *Supplementary Material*

## 1 SUPPLEMENTARY FIGURES

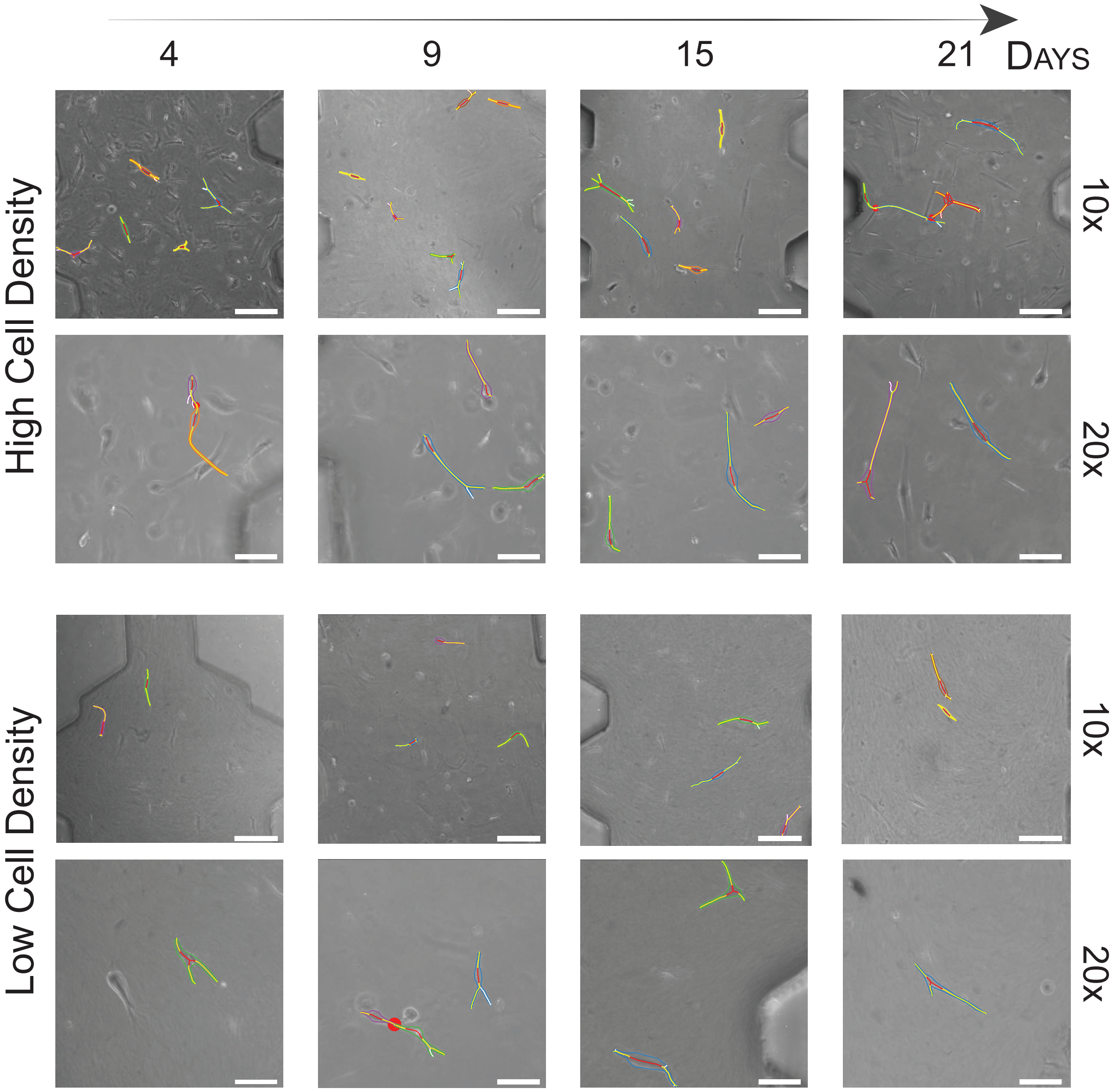


**Figure S1. Live image of primary human osteoblasts.** Cells cultured at low ($2.5 \times 10^5$ cells/ml) and high ($1 \times 10^6$ cells/ml) cell density. Scale bar for images magnified at 10x = 150 μm. Scale bar for images magnified at 20x = 75 μm

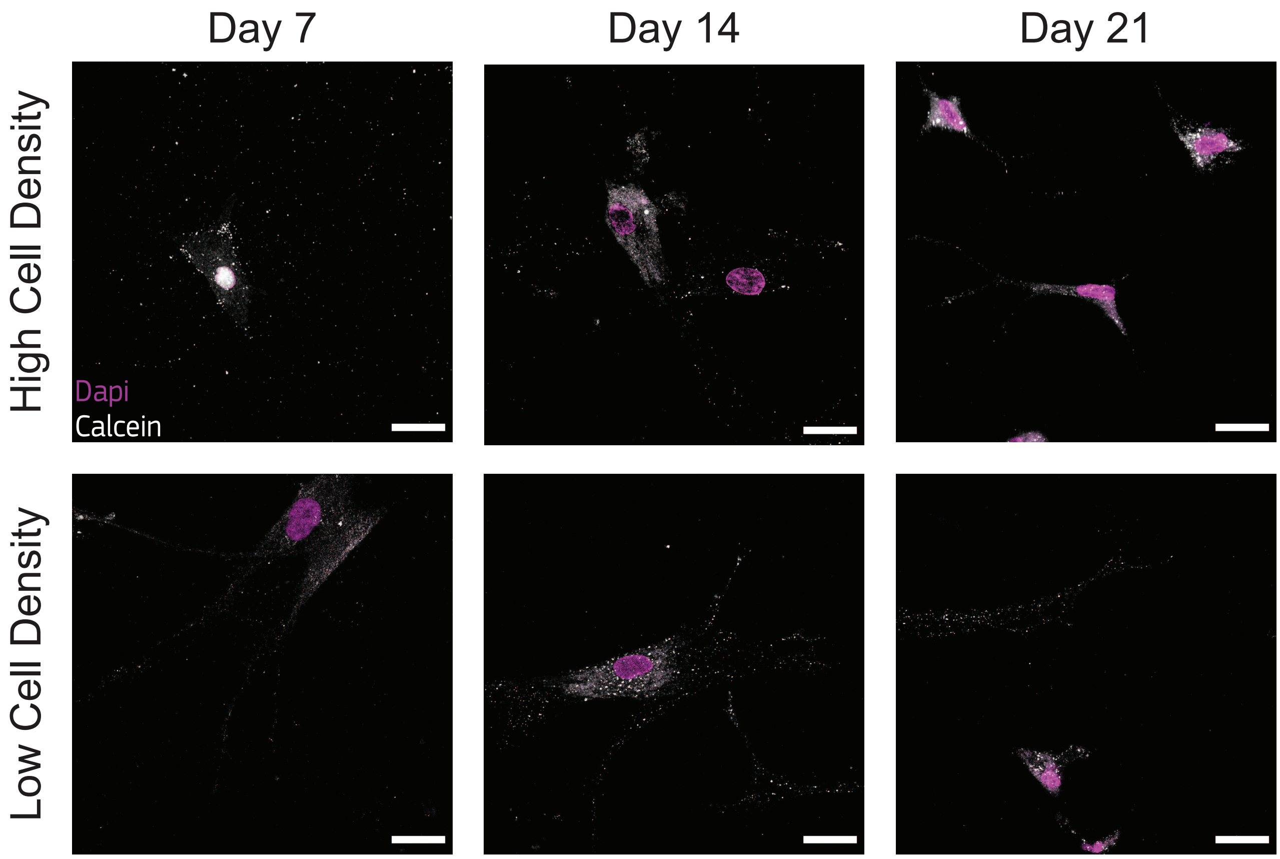


**Figure S2. Primary human osteoblasts cultured in the bone-on-a-chip device mineralize the 3D collagen matrix.** Representative confocal images of cell nuclei (DAPI) and calcium ions (calcein) bone-on-a-chip samples cultured at high and low cell density. Scale bar, 30 μm.
